# Perfluoroalkyl substances are associated with elevated blood pressure and hypertension in highly exposed young adults

**DOI:** 10.1186/s12940-020-00656-0

**Published:** 2020-09-21

**Authors:** Gisella Pitter, Maryam Zare Jeddi, Giulia Barbieri, Massimo Gion, Aline S. C. Fabricio, Francesca Daprà, Francesca Russo, Tony Fletcher, Cristina Canova

**Affiliations:** 1 Screening and Health Impact Assessment Unit, Azienda Zero-Veneto Region, Padova, Italy; 2 Unit of Biostatistics, Epidemiology and Public Health, Department of Cardio-Thoraco-Vascular Sciences and Public Health, Via Loredan 18, 35131 Padova, Italy; 3 Regional Center for Biomarkers, Department of Clinical Pathology, Azienda ULSS 3 Serenissima, Venice, Italy; 4 Laboratory Department-Regional Agency for Environmental Prevention and Protection-Veneto Region, Venice, Italy; 5 Directorate of Prevention, Food Safety, and Veterinary Public Health-Veneto Region, Venice, Italy; 6 London School of Hygiene and Tropical Medicine, London, UK

**Keywords:** Perfluoroalkyl substances, Blood pressure, Hypertension, Epidemiology, Cross-sectional study, Community exposure

## Abstract

**Background:**

Residents in a large area of North-Eastern Italy were exposed to perfluoroalkyl substances (PFAS) via drinking water. Studies on the association between PFAS and blood pressure levels are limited, and results are inconsistent. Using cross-sectional data from the Regional health surveillance program, we aimed to quantify the associations between PFAS serum concentrations and blood pressure and hypertension prevalence.

**Methods:**

The study comprised 16,224 individuals aged 20–39 years. Pregnant women (*n* = 327), or individuals with missing information on the selected covariates (*n* = 111) were excluded, leaving 15,786 subjects for the analyses. Hypertension was defined as any self-reported diagnosis, use of antihypertensive drugs, or elevated systolic blood pressure (SBP ≥ 140 mmHg)/diastolic blood pressure (DBP ≥ 90 mmHg). Generalized additive models were used to investigate the relation between perfluorooctanoic acid (PFOA), perfluorooctane sulfonic acid (PFOS), perfluorohexane sulfonic acid (PFHxS), and perfluorononanoic acid (PFNA)) natural log (ln) transformed and by decile, and SBP, DBP, hypertension, adjusted for potential confounders.

**Results:**

Both SBP and DBP increased significantly with an increase in the ln-transformed serum PFAS concentrations in a monotonic way. The predicted increase in SBP and DBP were 1.54 mmHg (95%CI 0.61–2.47), 1.60 mmHg (95%CI 0.92–2.27) from lowest to highest decile of PFOA. The associations were stronger for SBP in men and for DBP in women. One unit increase in each In-transformed PFAS was positively associated with an increased odd of hypertension in men: PFOA OR = 1.06 (1.01–1.11), PFOS OR = 1.13 (1.03–1.23), PFHxS OR = 1.08 (1.02–1.15), PFNA OR = 1.20 (1.02–1.40).

**Conclusions:**

Our findings suggest that serum PFAS concentrations were associated with increased systolic and diastolic blood pressure in a large highly exposed young adult population**.** Although the magnitude of the observed effect was relatively small, if confirmed it would be of public health relevance since even small increases in blood pressure levels at the population level may be associated to a raised risk of adverse outcomes such as cardiovascular disease and target organ damage.

## Background

Perfluoroalkyl substances (PFAS) are ubiquitous and highly persistent man-made chemicals widely used for a variety of commercial and industrial applications due to their grease-, stain, and water-repelling properties [1]. Consequently, human exposure to PFAS is widespread and mainly occurs through ingestion of contaminated food and dust [2]. Drinking water has been identified as a major source of exposure for many populations living near industrial and contaminated sites [3–6].

PFAS exposure has been associated with a number of risk factors of cardiovascular disease (CVD) including dyslipidemia, metabolic syndrome and thyroid disease [7]. High blood pressure is one of the most prevalent conditions increasing the risk of cardiovascular events [8]. Several studies in the general population [9–14], and just one on 500 highly exposed individuals in China [9] found positive associations between serum PFAS concentrations and hypertension. However, these findings were not demonstrated in other cross sectional studies [15–20], nor in two longitudinal studies on hypertension [21] and one on blood pressure trajectory [22]. The association of serum PFAS with blood pressure as a continuous outcome was evaluated in few cross-sectional studies, but with conflicting findings [9, 11, 12, 14].

Groundwater of a vast area of the Veneto Region (north-eastern Italy) was found to be contaminated by PFAS from a manufacturing plant active since the late 1960s. Residents were exposed to high concentrations of PFAS, particularly perfluorooctanoic acid (PFOA) through drinking water, until autumn 2013 [6]. A publicly funded health surveillance program is established to aid in the prevention, early diagnosis, and treatment of chronic disorders possibly associated with PFAS exposure. Using cross-sectional data from the regional health surveillance program we were able to model concentration-response curves over a wide range of internal doses thanks to the high dispersion of serum PFOA concentrations and the large sample size, enable us to model concentration-response curves over a wide range of internal doses. Therefore, we aimed to quantify the associations between PFAS serum concentrations and blood pressure and hypertension prevalence in a large population of highly exposed young adults. Moreover, we systematically evaluated gender-specific associations.

## Methods

### Participants and study design

The study consisted of 16,224 individuals aged 20–39 years recruited in the health surveillance program offered to the community of Veneto Region who was exposed for several decades to PFAS via drinking water distributed by contaminated public waterworks. The health surveillance program has been described in more detail elsewhere [6]. In brief, the target population was initially constituted by people born between 1951 and 2002 and residing in the municipalities that were identified as in the area served by PFAS-contaminated waterworks. Surveillance involved the active invitation of the eligible population and the free offer of health examinations including: I) a questionnaire on personal health history and lifestyle habits, socio-demographic characteristics, self-reported height and weight; II) measurement of blood pressure; and III) non-fasting blood and urine samples.

Pregnant women (*n* = 327), and individuals with missing information on the selected covariates (*n* = 111) were excluded, leaving in the analyses 15,786 subjects. No missing data on exposure and outcome variables were present (See Supplementary Figure 1, Additional File 1).

### PFAS exposure

Serum concentrations of twelve PFAS were measured by HPLC MS/MS (Shimadzu UFLC XR 20 Prominence coupled to Sciex API 4000): perfluorooctanesulfonate (PFOS), perfluorooctanoic acid (PFOA), perfluorohexanesulfonic acid (PFHxS), perfluorononanoic acid (PFNA), perfluoroheptanoic acid (PFHpA), perfluorobutanesulfonic acid (PFBS), perfluorohexanoic acid (PFHxA), perfluorobutanoic acid (PFBA), perfluoropentanoic acid (PFPeA), perfluorodecanoic acid (PFDeA), perfluoroundecanoic acid (PFUnA), and perfluorododecanoic acid (PFDoA). Details of the analytical method have been described elsewhere [6].

Method performances allow analytes to be detected as low as 0.1 ng/mL (LOD) and to be quantified above 0.5 ng/mL (LOQ). Only four PFAS quantifiable in at least 40% of samples were considered for the analyses: PFOA (detected in 99.86% of people), PFOS (detected in 99.70% of people), PFHxS (detected in 96.72% of people), PFNA (detected in 49.86% of people). Samples below the LOQ were assigned a value equal to LOQ/√2. The most extreme outliers of PFAS serum levels were removed as followings: (PFOA> 700 mg/L (*n* = 6), PFOS > 50 mg/L (*n* = 9), PFHxS> 100 mg/L (*n* = 3), PFNA > 10 mg/L (*n* = 1)).

### Blood pressure and hypertension

Blood pressure (BP) was measured by trained nurses with participants first sitting at rest for at least five minutes, according to the European Society of Hypertension recommendations [23]. A validated semi-automatic sphygmomanometer with an appropriate cuff size for the arm circumference was used. When the first measure was ≥140 mmHg for systolic blood pressure (SBP) or ≥ 90 mmHg for diastolic blood pressure (DBP), a second measurement was taken at least two minutes apart. In general, 1714 subjects went through the second measurement. When the second measurement was within the cut-offs, the second measurement was used (*n* = 1078). Otherwise, the mean of the two measurements was considered when both measurements were above the cut-offs (*n* = 636).

Medical history data were collected directly from participants by trained nurses via structured software-based questionnaire using in-person interviews at the study enrolment. The questionnaire included items on personal health history (“Which diseases do you suffer from?”) and medications (“Do you take any medication on a regular basis?” “If yes, which medications do you take?”).

Hypertension was defined considering any self-reported diagnosis of hypertension, reported use of antihypertensive medications, or raised SBP (≥140 mmHg) or DBP (≥90 mmHg).

### Covariates

We obtained information on age, gender, country of birth, education level, smoking habits, body mass index (BMI), physical activity, history of certain diseases, medication, alcohol consumption, and food intakes including salt habit. Standard data checks and cleaning procedures (e.g. range and consistency checks) were used to minimize errors and missing values and to maximize data quality. Data on food consumption were transformed from number of serving per day/week/month to number of serving per week for all the food categories to create harmonized diet pattern classification. After checking the accuracy of data on BMI regarding height and weight, BMI was recalculated and classified as underweight (< 18.5 kg/m^2^), normal weight (18.5–24.9 kg/m^2^), overweight (25–29.9 kg/m^2^), obese (≥30 kg/m^2^). Alcohol consumption was categorized in 0, 1–2, 3–6, 7+ alcohol units per week. Smoking status was subdivided into current smokers, previous smokers and non-smokers. Degree of physical activity (Light, Moderate, or Heavy) was defined based on an algorithm that combined information reported by the subject on intensity, duration, and frequency of all types of physical activity practiced during the week [6]. Countries of birth were classified in two categories based on geographical areas including: Italy plus other Highly Developed Countries, and High Migratory Pressure Countries. The time-lag between the beginning of the study (1st January 2017) and the date of enrollment was calculated for each subject and included as possible covariate (number of months). Information on the center in charge of the BP measurements was considered as possible confounder in statistical analyses.

Covariates to be included as potentially confounders of the BP/PFAS association were selected from the available variables, based on related literature, through the construction of a directed acyclic graph (DAG) representing the identification of a minimally sufficient set of variables to control confounding. The minimally sufficient adjustment set was identified using DAGitty v1.0 (www.dagitty.net) implemented in R (R Development Core Team 2010, R Foundation for Statistical Computing, Vienna, Austria. ISBN 3–900,051–07-0, URL: http://www.R-project.org/).

### Statistical analysis

The serum concentrations of PFAS by gender were expressed as arithmetic mean, standard deviation (SD) and percentiles. Since data on PFAS were markedly skewed to the right, concentrations were natural log (ln) transformed in order to improve normality of the data distribution. Spearman’s correlation (ρ) was used to describe pair-wise relations between the PFAS.

Our main outcomes are continuous SBP and DBP. For these analyses, participants with self-reported diagnosis of hypertension or under treatment with antihypertensive medications (*n* = 406) were excluded, leaving 15,380 subjects in analysis (See Supplementary Figure 1, Additional File 1).

We used generalized additive models (GAMs) to analyze the relation between each (ln) PFAS and BP outcomes, adjusted for the potential set of confounders. In order to explore the shape of possible associations between PFAS and BP levels the models used thin plate spline smooth terms [24] for the exposures and continuous covariates, and plotting the predicted values. Degree of smoothing was selected by generalized cross validation as implemented in the R package mgcv [25]. Since the spline analysis showed associations compatible with a linear relationship on the ln PFAS, linear regression coefficient (β) and 95% confidence intervals (CI) were reported. Serum PFAS levels were also categorized into quartiles, in order to limit the influence of extreme values, with the exception of PFNA for which the large proportion below the LOQ did not allow the quartiles subdivision.

For the analyses on PFAS associations with hypertension prevalence, a binomial link function was used in the models and Odds Ratios (ORs) were calculated, together with their 95% confidence intervals (95%CI).

All analyses were fully adjusted for the established set of covariates: age, BMI, time-lag between the enrolment and the beginning of the study (all continuous variables modelled using thin plate spline) and categorical covariates including gender, physical activity, smoking habits, food consumption (tertiles or quartiles of fruit/vegetables, milk/yogurt, cheese, meat, sweet/snacks/sweet beverage, eggs, fish, bread/pasta/cereals per week), salt habit, country of birth, alcohol consumption, education level and center in charge of the BP measurement (Lonigo, Legnago, San Bonifacio, and Noventa Vicentina).

All the above analyses have been also stratified according to gender and an interaction term between gender and ln-PFAS was also added to the main models.

Since PFAS are predominantly excreted by the kidney through glomerular filtration and impaired kidney function is associated with raised BP [23], to assess for possible confounding a sensitivity analysis was conducted adjusting all models for estimated glomerular filtration rate (eGFR with cut-off < 90 mL/min) calculated according to the CKD-EPI equation [26].

Finally, we analysed SBP and DBP associations with PFAS excluding subjects with raised BP (SBP ≥ 140 mmHg or DBP ≥90 mmHg).

The procedures of the health surveillance program changed over time: until 31 December 2017, blood sampling, and interview and BP measurement were carried out in the same session for each participant, whilst thereafter they were performed in two different sessions roughly 1 month apart in order to be able to provide blood test results on the day the participant came for the interview and the BP measurement. To explore whether this organizational change may have affected the PFAS/BP associations, analyses were also restricted to the subgroup of 10,656 individuals recruited after 31 December 2017.

The level of statistical significance was set at 0.05. The statistical software STATA/SE version 13.0 (Stata Corp LP, College Station, TX, USA) and R (R Development Core Team 2010, R Foundation for Statistical Computing, Vienna, Austria. ISBN 3–900,051–07-0, URL: http://www.R-project.org/) was used for statistical analyses.

## Results

Table 1 provides descriptive results on PFAS, outcome variables and selected covariates of the study population. 7667 (49%) males and 8119 (51%) females were included in the analyses with a mean age of 30 years (SD 5.9). Further characteristics of participants according to gender are reported in Supplementary Table 1, Additional File 2.

**Table 1:** Characteristics of the study population (*n* = 15,786), stratified by gender

Variables	Total (*n* = 15,786)			Males (***n*** = 7667)			Females (***n*** = 8119)		
	mean (SD)	min-max	Median (Q1-Q3)	mean (SD)	min-max	Median (Q1-Q3)	mean (SD)	min-max	Median (Q1-Q3)
**PFAS**									
PFOA (ng/mL)	59.69 (72.26)	0.35–1400	35.8 (13.7–78.9)	83.84 (87.51)	0.35–1400	58.3 (25.1–114.7)	36.89 (43.03)	0.35–671	22.6 (8.8–49.4)
PFOS (ng/mL)	4.63 (4.02)	0.35–142	3.7 (2.5–5.6)	5.72 (4.43)	0.35–142	4.8 (3.3–6.9)	3.59 (3.27)	0.35–124	3 (2–4.4)
PFHxS (ng/mL)	5.97 (6.82)	0.35–127	3.6 (1.6–7.8)	8.86 (8.29)	0.35–127	6.5 (3–12)	3.24 (3.19)	0.35–41.3	2.2 (1.1–4.3)
PFNA (ng/mL)	0.53 (0.42)	0.35–39.7	0.35 (0.35–0.6)	0.59 (0.52)	0.35–39.7	0.5 (0.35–0.7)	0.48 (0.28)	0.35–8.8	0.35 (0.35–0.5)
**Covariates**									
Age (years)	30.04 (5.86)	20–39	30 (25–35)	29.88 (5.88)	20–39	30 (25–35)	30.2 (5.84)	20–39	31 (25–35)
BMI (kg/m^2^)	23.91 (4.3)	13.58–50.47	23.27 (20.9–26.04)	24.75 (3.82)	13.58–49.01	24.22 (22.23–26.59)	23.11 (4.56)	14.32–50.47	22.04 (19.92–25.08)
Time-lag (months)	14.77 (5.45)	3–28	14 (10–19)	14.7 (5.51)	5–28	14 (10–19)	14.84 (5.39)	3–28	14 (10–19)
Gender	**n (%)**								
Male	7667 (48.57)								
Female	8119 (51.43)								
Education	**n (%)**			**n (%)**			**n (%)**		
Elementary/Middle school	2409 (15.26)			1346 (17.56)			1063 (13.09)		
Highschool	9445 (59.83)			4847 (63.22)			4598 (56.63)		
University	3932 (24.91)			1474 (19.23)			2458 (30.27)		
Physical activity	**n (%)**			**n (%)**			**n (%)**		
Light	10,638 (67.39)			4719 (61.55)			5919 (72.9)		
Moderate	2394 (15.17)			1152 (15.03)			1242 (15.3)		
Heavy	2754 (17.45)			1796 (23.43)			958 (11.8)		
Smoke	**n (%)**			**n (%)**			**n (%)**		
NO	9230 (58.47)			3895 (50.8)			5335 (65.71)		
YES	4355 (27.59)			2575 (33.59)			1780 (21.92)		
Ex	2201 (13.94)			1197 (15.61)			1004 (12.37)		
Country of birth	**n (%)**			**n (%)**			**n (%)**		
HDC	14,297 (90.57)			7136 (93.07)			7161 (88.2)		
HMPC	1489 (9.43)			531 (6.93)			958 (11.8)		
**Outcomes**									
	**n (%)**			**n (%)**			**n (%)**		
Hypertension	1971 (12.49)			1449 (18.9)			522 (6.43)		
	**Total (*n*** **= 15,380)**			**Males (*n*** **= 7428)**			**Females (*n*** **= 7952)**		
Blood pressure	**mean (SD)**	**min-max**	**Median (Q1-Q3)**	**mean (SD)**	**min-max**	**Median (Q1-Q3)**	**mean (SD)**	**min-max**	**Median (Q1-Q3)**
Systolic BP (mmHg)	119.77 (14.23)	70–180	120 (110–130)	125.08 (13.51)	70–180	125 (118–134)	114.8 (13.04)	70–180	115 (105–122)
Diastolic BP (mmHg)	74.36 (9.74)	25–119	75 (70–80)	76.36 (9.58)	25–119	79 (70–81)	72.51 (9.51)	40–112	72 (65–80)

Among the four PFAS, PFOA was detected at the highest level (median 35.8 ng/mL), followed by PFOS (median 3.7 ng/mL), PFHxS (median 3.6 ng/mL), and PFNA (median 0.35 ng/mL). The highest serum levels of all PFAS were found in males compared to females. Moderate to strong correlations were observed among the measured PFAS. The most highly correlated compounds were PFHxS with PFOA (ρ = 0.91); while the least correlated were PFNA with PFOA and PFHxS (both ρ = 0.40). The Spearman correlation coefficients of PFOS with PFOA and PFHxS were 0.63 and 0.68, respectively.

Hypertension was detected in 1971 (12.5%) of the recruited subjects (19% in males and 6% in females). Significant differences in demographic and lifestyle characteristics according to hypertensive status were observed among participants (See Supplementary Table 2, Additional File 3). In particular, hypertensive subjects were older, had a lower educational level, and a higher BMI. Moreover, significant differences in the prevalence of hypertension were found between centers in charge for blood pressure measurement.

Table 2 depicts the results of the fitted models assessing the association between each PFAS, considered as ln-linear predictors or categorical predictors (based on quartiles), and SBP and DBP, after adjustment for confounding factors. As shown in Table 2, each ln-increase in PFOA was associated with an increase in SBP of 0.37 mmHg (95%CI 0.19–0.54), 0.57 mmHg (95%CI 0.24–0.90) for PFOS, 0.37 mmHg (95%CI 0.15–0.58) for PFHxS, and 0.99 mmHg (95%CI 0.47–1.51) for PFNA. Similarly, each ln-increase in PFOA was associated with an increase in DBP of 0.34 mmHg (95%CI 0.21–0.47), 0.44 mmHg (95%CI 0.20–0.68) for PFOS, 0.33 mmHg (95%CI 0.17–0.48) for PFHxS, and 0.62 mmHg (95%CI 0.24–1.00) for PFNA. Subjects in the highest PFOA quartile had respectively 1.07 mmHg (95%CI 0.46–1.68) and 0.97 mmHg (95%CI 0.53–1.42) higher SBP and DBP than those in the lowest quartile. Slightly lower increments were seen for the other PFAS. The predicted increase in SBP and DBP were 1.54 mmHg (95%CI 0.61–2.47), 1.60 mmHg (95%CI 0.92–2.27) from lowest to highest decile of PFOA and 1.25 mmHg (95%CI 0.36–2.14), 1.02 mmHg (95%CI 0.37–1.67) from lowest to highest decile of PFOS (See Supplementary Figure 2, Additional File 4).

**Table 2:** Association between PFAS (ln ng/mL) and systolic and diastolic blood pressure (mmHg from GAM models (*n* = 15′380), adjusted by several covariates: β coefficients and 95% Confidence Intervals (CI)

PFAS	Systolic Blood Pressure						Diastolic Blood Pressure					
	Total		Males		Females		Total		Males		Females	
	β	CI 95%	β	CI 95%	β	CI 95%	β	CI 95%	β	CI 95%	β	CI 95%
ln_PFOA	**0.37**	0.19–0.54	**0.46**	0.19–0.73	**0.31**	0.08–0.55	**0.34**	0.21–0.47	**0.23**	0.04–0.42	**0.39**	0.21–0.57
IQ	**113.81**		**122.08**		**113.09**		**73.27**		**77.4**		**72.38**	
II Q	0.26	−0.29 - 0.81	0.12	−0.87 - 1.1	0.46	−0.2 - 1.13	0.24	−0.16 - 0.64	−0.31	−1 - 0.38	0.44	−0.06 - 0.94
III Q	**0.74**	0.16–1.31	0.8	−0.15 - 1.75	**0.8**	0.06–1.53	**0.78**	0.36–1.2	0.4	− 0.27 - 1.07	**0.74**	0.19–1.3
IV Q	**1.07**	0.46–1.68	**1.25**	0.32–2.18	0.71	− 0.2 - 1.62	**0.97**	0.53–1.42	0.49	− 0.17 - 1.14	**1.15**	0.46–1.83
ln_PFOS	**0.57**	0.24–0.9	**0.98**	0.47–1.48	0.32	−0.13 - 0.77	**0.44**	0.2–0.68	0.29	− 0.07 - 0.64	**0.51**	0.17–0.84
IQ	**114.15**		**122.21**		**114.34**		**73.51**		**77.51**		**72.67**	
II Q	−0.01	−0.56 - 0.53	0.46	−0.55 - 1.47	− 0.07	−0.72 - 0.58	0.32	−0.08 - 0.72	0.3	−0.41 - 1.01	0.24	−0.24 - 0.73
III Q	0.27	−0.29 - 0.84	0.79	−0.18 - 1.76	0.26	−0.47 - 0.98	0.3	−0.12 - 0.71	0.12	−0.57 - 0.8	0.26	−0.29 - 0.8
IV Q	0.6	0–1.21	**1.13**	0.16–2.09	0.42	−0.45 - 1.29	**0.57**	0.13–1.02	0.19	−0.49 - 0.87	**1.00**	0.34–1.66
ln_PFHxS	**0.37**	0.15–0.58	**0.61**	0.3–0.93	0.17	− 0.13 - 0.47	**0.33**	0.17–0.48	**0.29**	0.07–0.51	**0.26**	0.04–0.49
IQ	113.983		**121.71**		**113.4**		**73.37**		**77.03**		**72.65**	
II Q	−0.01	−0.56 - 0.54	0.63	−0.43 - 1.69	− 0.14	−0.78 - 0.51	0.2	−0.2 - 0.6	0.49	−0.25 - 1.24	0.04	−0.44 - 0.52
III Q	**0.61**	0.04–1.18	**1.16**	0.14–2.17	0.57	−0.14 - 1.28	**0.7**	0.28–1.11	**0.74**	0.03–1.46	**0.57**	0.03–1.11
IV Q	**0.93**	0.3–1.57	**1.54**	0.55–2.53	0.43	−0.61 - 1.46	**0.78**	0.32–1.25	**0.75**	0.05–1.45	0.75	−0.03 - 1.53
ln_PFNA	**0.99**	0.47–1.51	**1.46**	0.72–2.19	0.54	−0.21 - 1.28	**0.62**	0.24–1	0.36	− 0.16 - 0.88	**0.81**	0.25–1.37

**Fig. 1:**
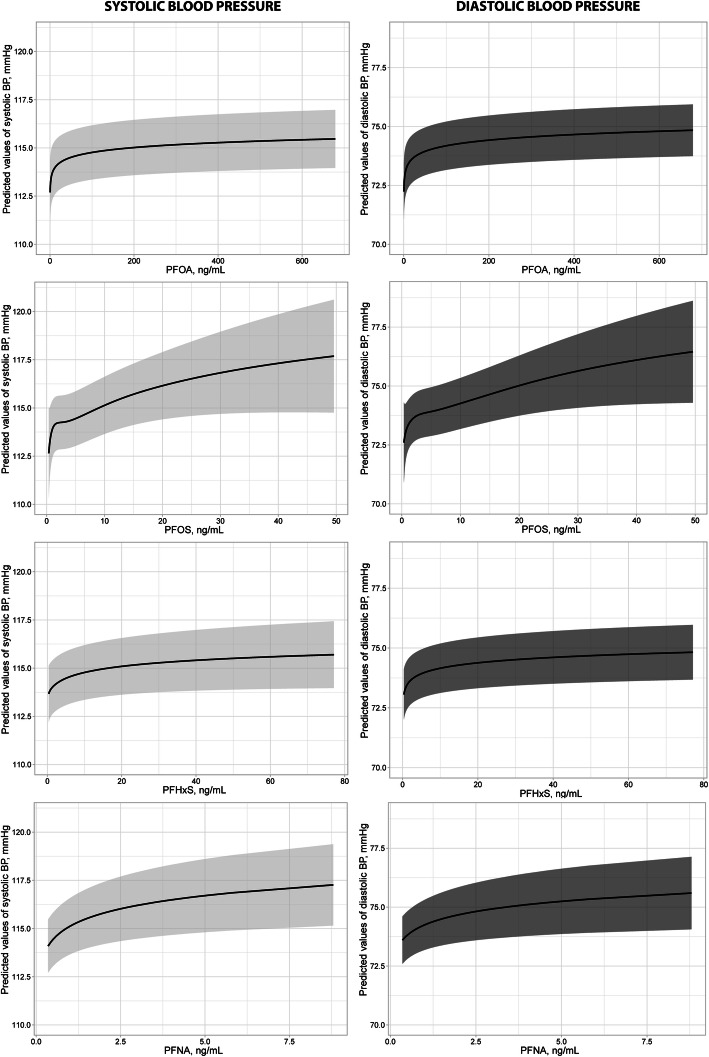
Exposure–response curves for PFAS exposure and Systolic and Diastolic Blood pressure from GAM models using thin plane splines, with 95% confidence intervals. The predicted levels are based on average characteristics used as covariates in the models

Furthermore, the adjusted concentration-response curves between SBP, DBP and PFAS in the total studied population are shown in Fig. 1 and the results stratified by gender are presented in the Supplementary Figure 3, Additional File 5. These analyses revealed highly significant associations between both SBP and DBP and all investigated PFAS with approximately linear relationships between the natural logarithm of the four PFAS and these outcomes, thus showing a linear-log relationship with the back transformed PFAS values (See Supplementary Table 3, Additional File 6).

Gender significantly modified the association between all PFAS and SBP, with significant associations seen almost exclusively among men; on the contrary, women showed stronger associations with DBP (although with no significant *p*-value for interaction). Adjusting for eGFR did not change the results of the observed associations between PFAS and SBP or DBP (See Supplementary Table 4, Additional File 7). The analyses on the restricted population recruited after 31 December 2017 also showed similar results (See Supplementary Table 5, Additional File 8) as the one limited to those subjects with SBP and DBP within normal ranges (See Supplementary Table 6, Additional File 9).

Table 3 presents results of multivariable logistic models for the association between PFAS levels as ln-linear predictors or categorical predictors (based on quartiles) and hypertension prevalence. One unit increase in each ln-PFAS was positively associated with an increased odds of hypertension after controlling for potential confounders: PFOA OR = 1.06 (95%CI 1.01–1.12), PFOS OR = 1.12 (95%CI 1.02–1.22), PFHxS OR = 1.08 (95%CI 1.02–1.15), PFNA OR = 1.10 (95%CI 0.96–1.26). When stratified by gender, positive associations were detected only in men, with the strongest effect seen for PFNA OR = 1.19 (95%CI 1.02–1.40) (*p*-value interaction = 0.048).

**Table 3:** Association between PFAS and risk of hypertension from GAM models adjusted by several covariates, using PFAS quartiles (n = 15,786): odds ratios (OR) and 95% Confidence Intervals (CI)

PFAS	Total			Males			Females		
	OR	CI Lower	CI Upper	OR	CI Lower	CI Upper	OR	CI Lower	CI Upper
**ln_PFOA**	**1.06**	1.01	1.12	**1.08**	1.02	1.15	1.06	0.97	1.15
IQ	1			1			1		
II Q	1.00	0.85	1.16	1.00	0.81	1.24	1.05	0.84	1.32
III Q	1.02	0.87	1.20	1.11	0.90	1.36	0.93	0.71	1.23
IV Q	1.16	0.99	1.37	1.21	0.99	1.48	1.17	0.84	1.62
**ln_PFOS**	**1.12**	1.02	1.22	**1.17**	1.05	1.31	1.06	0.91	1.24
IQ	1			1			1		
II Q	0.99	0.85	1.16	1.10	0.88	1.37	0.97	0.77	1.22
III Q	1.06	0.91	1.24	1.26	1.02	1.55	**0.85**	0.65	1.12
IV Q	1.12	0.95	1.32	1.28	1.03	1.58	1.02	0.74	1.41
**ln_PFHxS**	**1.08**	1.02	1.15	**1.11**	1.03	1.19	1.08	0.97	1.20
IQ	1			1			1		
II Q	1.01	0.86	1.19	1.08	0.85	1.37	1.01	0.80	1.26
III Q	1.08	0.92	1.27	1.19	0.95	1.50	1.00	0.77	1.30
IV Q	1.19	1.00	1.41	**1.27**	1.02	1.59	1.28	0.90	1.83
**ln_PFNA**	1.10	0.96	1.26	**1.19**	1.02	1.40	0.94	0.71	1.25

## Discussion

In the present cross-sectional study consisting of more than 15,000 young adults from a highly exposed community, serum PFAS concentrations displayed a positive association with blood pressure levels and were also associated with a raised prevalence of hypertension in men. The magnitude of the association with blood pressure was relatively small and for/regarding systolic blood pressure, was significantly modified by gender, with associations seen almost only for men.

To the best of our knowledge, this is the first study that formally investigated the shape of the association between serum PFAS and blood pressure in a large population of highly exposed community residents (more than 15,000 participants) consisting of a homogeneous age group of young adults from 20 to 39 years old. Literature on the association between PFAS exposure and blood pressure and/or hypertension (defined as self-reported diagnosis or treatment or elevated measured blood pressure) is relatively limited, with contradictory findings. Some large cross-sectional studies were conducted on representative samples of the US general population with background exposure using data from the NHANES survey [11, 12, 14, 18, 19]. Among 2934 adults, Min et al. showed a significant increase in SBP, but not DBP, associated with increasing PFOA concentrations [12]. Moreover, the Odds Ratio of hypertension was 2.62 when comparing the 80th and 20th percentiles of the PFOA distribution. In contrast, He et al. (*n* = 7543) reported no significant association with blood pressure, but blood pressure was not the primary outcome of this study [19]. In a recent cross-sectional study on 6967 adults from NHANES, Liao et al. found a positive log-linear association of serum PFOA, PFOS, and PFHxS with SBP and a non-linear J-shaped association of serum PFOS and PFNA with DBP; moreover, the study showed a non-linear J-shaped association of serum PFOA and PFNA with the odds of hypertension [14]. The other two studies focused on children and adolescents with background exposure to PFAS [11, 18], yielding conflicting results: while Geiger et al. (*n* = 1655) found no evidence of an association between serum PFAS and blood pressure or hypertension [18], Ma et al. (*n* = 2251) found a positive significant association between PFOS and DBP among males [11]. Other cross-sectional studies with smaller sample size (range *n* = 48–187) reported positive [10, 13], null [15, 17] or negative associations [16]. A study on highly exposed Chinese adults (China C8 Health Project, *n* = 1612) investigated the association between blood pressure and several PFAS congeners, including linear and branched isomers [9]. Significant positive associations emerged for log-transformed PFOS, PFOA, PFNA, PFBA, and PFPeA, but not PFHxS. As for PFOA, the association was attributable only to branched isomers. Moreover, a strong gender difference was found, with more significant associations among females [9].

To our knowledge, only two studies assessed the association between PFAS exposure and blood pressure or hypertension with a longitudinal design [21, 22]. In the C8 Health Project, a large study involving a highly exposed community of the Mid-Ohio Valley, USA, there was no clear evidence of an association between PFOA exposure and self-reported incident hypertension, although small statistically significant associations emerged in certain subgroups (females aged 20–39 years and males aged 40–59 years) [21]. Lin and colleagues explored the issue using data from a randomized controlled trial involving 957 pre-diabetic patients that underwent either a lifestyle intervention or standard care. At baseline, they found a positive cross-sectional association between PFOA serum levels and SBP and a significantly increased prevalence of hypertension with higher serum PFOA among males. In the prospective analyses, however, the associations with both blood pressure trajectories and the risk of developing hypertension were null, except for an inverse association between PFOS and SBP in the intervention arm [22].

As outlined above, the available literature shows several inconsistencies: the direction, statistical significance, and magnitude of associations, as well as the gender effect modification, were different among studies. This may be attributable to the heterogeneous characteristics of the investigated populations, in terms of age, social context, and levels of exposure. Moreover, reported effect sizes are poorly comparable with each other. The two studies more comparable with the present one in terms of design and population size were conducted on the general US population with exposure levels quite different from ours [9, 12]. Min et al. reported median serum PFOA of 4 mcg/L, and Bao et al. reported median serum PFOA of 6.19 ng/ml and median serum PFOS of 24.22 ng/ml. Both studies found associations of greater magnitude than ours. Min and colleagues reported linear regression coefficients between log-transformed serum PFOA and SBP of 2.48 and 0.75 for DBP. In the study by Bao et al., for each ln-unit increase of serum PFOA, PFOS, and PFNA, SBP increased by 1.69, 4.84, and 3.01 mmHg, and DBP increased by 2.18, 2.70, and 2.48 mmHg, respectively.

Although the magnitude of the observed effect was relatively small, if confirmed it would be of public health relevance since the greatest magnitude of effect was observed at lower serum PFAS concentrations corresponding to background exposure and it is widely recognized that even small increases in blood pressure levels imply a raised risk of adverse outcomes such as CVD and target organ damage [27]. It has been estimated that a 1 mmHg-reduction of SBP at the population level may be associated to a decrease in the incidence rate for 100,000 person-years of 9 coronary heart disease events, 4.8 stroke events, and 13.3 heart failure events [28]. Of note, the detrimental long-term effects of blood pressure elevation have been demonstrated also in young adults [29]. Nevertheless, only few studies have investigated the association between PFAS and CVD so far, with inconsistent findings [21, 30–33]. In the C8 Health Study, cumulative serum PFOA was associated with a significantly increased risk of stroke with a non-monotonic dose-response relationship [33]. The association with the risk of coronary artery disease was less clear since it was present only in males of certain age subgroups [21].

The biological mechanisms underlying the association between PFAS exposure and elevated blood pressure have still to be fully elucidated. Potential biological pathways may include oxidative stress leading to impaired vasodilation [34–37]. Oxidative stress is proposed to play a critical role in the pathogenesis of hypertension [38, 39]. Moreover, intra-uterine exposure has been shown to reduce the number of nephrons at birth [40], which may be associated with over-activation of existing nephrons, pressure natriuresis, and blood pressure elevation. This hypothesis would be compatible with our findings since our study population was composed of young adults that may have been exposed to PFAS since conception. Furthermore, PFAS might interfere with the signaling pathways of the thyroid hormones [7] which have an important role in energy metabolism and blood pressure regulation. Therefore, PFAS may influence blood pressure with a combination of various physiological processes such as alteration of the levels of thyroid hormones or level of total cholesterol as indirect mechanism of actions [41]. Furthermore, PFOS as potential endocrine disrupting chemical was also related to the transcriptional induction of the gene cyp11b2 (encoding CYP11B2, aldosterone synthetase). Aldosterone is involved in blood pressure regulation by increasing the reuptake of ions and water in the kidneys [42].

The observed gender differences were inconsistent between studies [9, 11] and require further investigations. The fact that in our study the association with SBP and hypertension was present mainly in men may reflect the lower serum PFAS levels and lower prevalence of hypertension among women. Women had a higher proportion of PFAS levels below the quantification limit, which makes more difficult to know the real PFAS levels in this subgroup. The half-life difference in males and females [43] may be one of the underlying factors affecting the difference in the results according to gender. Those differences may also be related to the effect of sex hormones on blood pressure regulation in a young adult population like the one of the present study. In one hand, estradiol has been shown to promote peripheral vasodilation with ensuing lower blood pressure, and on the other hand menopause is associated to an increase of blood pressure levels compared to the reproductive age [44]. Some in vitro studies indicate that PFAS may interfere with the signaling of human sex hormones [45–47], which may also contribute to the observed gender differences of the association between PFAS and blood pressure.

Strengths of this study include the large population and the availability of an accurate measure of the internal dose of PFAS and of information on several anthropometric, lifestyle, and clinical variables, that allowed adjustment for many possible confounders. The high dispersion of serum PFOA concentrations in our population allowed to model concentration-response curves over a wide range of internal doses, covering both background and high exposure levels. Moreover, we systematically evaluated gender-specific associations. The main limitation is the cross-sectional design that precludes evaluation of the temporal relationship between exposure and outcome. A limitation in looking at individual PFAS is that they are highly correlated. Especially for the compounds with lower serum levels, such as PFNA in our study, the association may reflect associations driven by the correlated PFAS at higher concentration. The high correlation also prevents multipollutant models to assess their mutual adjustment. Also, we were unable to investigate the role of different PFAS isomers since only total PFAS serum concentrations were available. Another important limitation is that in most subjects we relied on a single blood pressure measurement. Blood pressure is subject to high intra-individual variation, therefore a single measurement may be poorly representative of an individual's mean blood pressure [23]. Moreover, results of blood pressure measurements are both device- and operator-dependent [48]. Indeed, we observed a significant difference of hypertension prevalence between centers in charge for blood pressure measurement. Random intra-individual variation would cause a non-differential misclassification of outcome and therefore would bias associations towards the null. By contrast, device- and operator-dependence may have introduced a systematic misclassification; however, to control for this we adjusted all analyses by center in charge for blood pressure measurement. The change in the procedures occurred after December 2017 may have provoked a differential misclassification of outcome in the case participants were informed of their serum PFAS levels prior to blood pressure measurement, possibly resulting in higher blood pressure among subjects with higher PFAS levels due to alarm reaction. To address this issue, we restricted analyses to participants recruited after 31 December 2017, but we found no evidence of such a systematic error since results were very similar to the overall analyses.

## Conclusions

In conclusion, our findings suggest that serum PFAS concentrations are associated with raised blood pressure levels with the greatest increases seen at the lower internal dose range. These are cross-sectional associations and should be interpreted with caution and the average change in blood pressure is quite modest. However, PFAS exposure is widespread and so if the association is causal, it would imply a significant population health impact. Such findings should be confirmed by further studies with a longitudinal design and more refined methods to measure blood pressure levels. Moreover, longitudinal studies are warranted to assess whether PFAS exposure is a risk factor for the development of cardiovascular events.

## Supplementary information

**Additional file 1: Figure** **1.** Flowchart of the study population.

**Additional file 2: Table** **1.** Descriptive statistics of diet and center where Blood Pressure has been measured.

**Additional file 3: Table** **2.** Descriptive statistics in subjects with or without hypertension, stratified by gender.

**Additional file 4: Figure 2.** Exposure–response curves for PFAS exposure and Systolic and Diastolic Blood pressure from GAM models using thin plane splines, with 95% confidence intervals, stratified by gender. The predicted levels are based on average characteristics used as covariates in the models.

**Additional file 5: Figure 3.** Predicted values of Systolic and Diastolic Blood Pressure by deciles of PFAS distribution.

**Additional file 6: Table** **3.** GAM model with using PFAS thin plate spline smooth terms: EDF and *p*-values.

**Additional file 7: Table 4.** GAM models adjusted by eGFR (cut-off 90 ml/min).

**Additional file 8: Table 5.** GAM models on a restricted population recruited after December 2017 (*n* = 10,656).

**Additional file 9: Table 6.** GAM models on a restricted population, excluding subclinical hypertensive (*n* = 13,815).

## Data Availability

Data contains sensitive personal information and cannot be made publicly available. Any data inquiries are referred to the corresponding author (cristina.canova@unipd.it).

## Abbreviations

PFAS: Perfluoroalkyl substances
SBP: Systolic blood pressure
DBP: Diastolic blood pressure
PFOS: Perfluorooctanesulfonate
PFOA: Perfluorooctanoic acid
PFHxS: Perfluorohexanesulfonic acid
PFNA: Perfluorononanoic acid
PFHpA: Perfluoroheptanoic acid
PFBS: Perfluorobutanesulfonic acid
PFHxA: Perfluorohexanoic acid
PFBA: Perfluorobutanoic acid
PFPeA: Perfluoropentanoic acid
PFDeA: Perfluorodecanoic acid
PFUnA: Perfluoroundecanoic acid
PFDoA: Perfluorododecanoic acid
LOD: Limit of detection
LOQ: Limit of quantification
BP: Blood pressure
BMI: Body mass index
DAG: Directed acyclic graph
ln-PFAS: Natural log (ln) transformed PFAS
GAMs: Generalized additive models
eGFR: Estimated glomerular filtration rate
