# Supplementary figures and images for: Perfluoroalkyl substances are associated with elevated blood pressure and hypertension in highly exposed young adults
Source: Environ Health. 2020 Sep 21;19:102. doi: 10.1186/s12940-020-00656-0 (PMC7507812; doi:10.1186/s12940-020-00656-0)

**Additional File 1**

Figure 1. Flowchart of the study population.


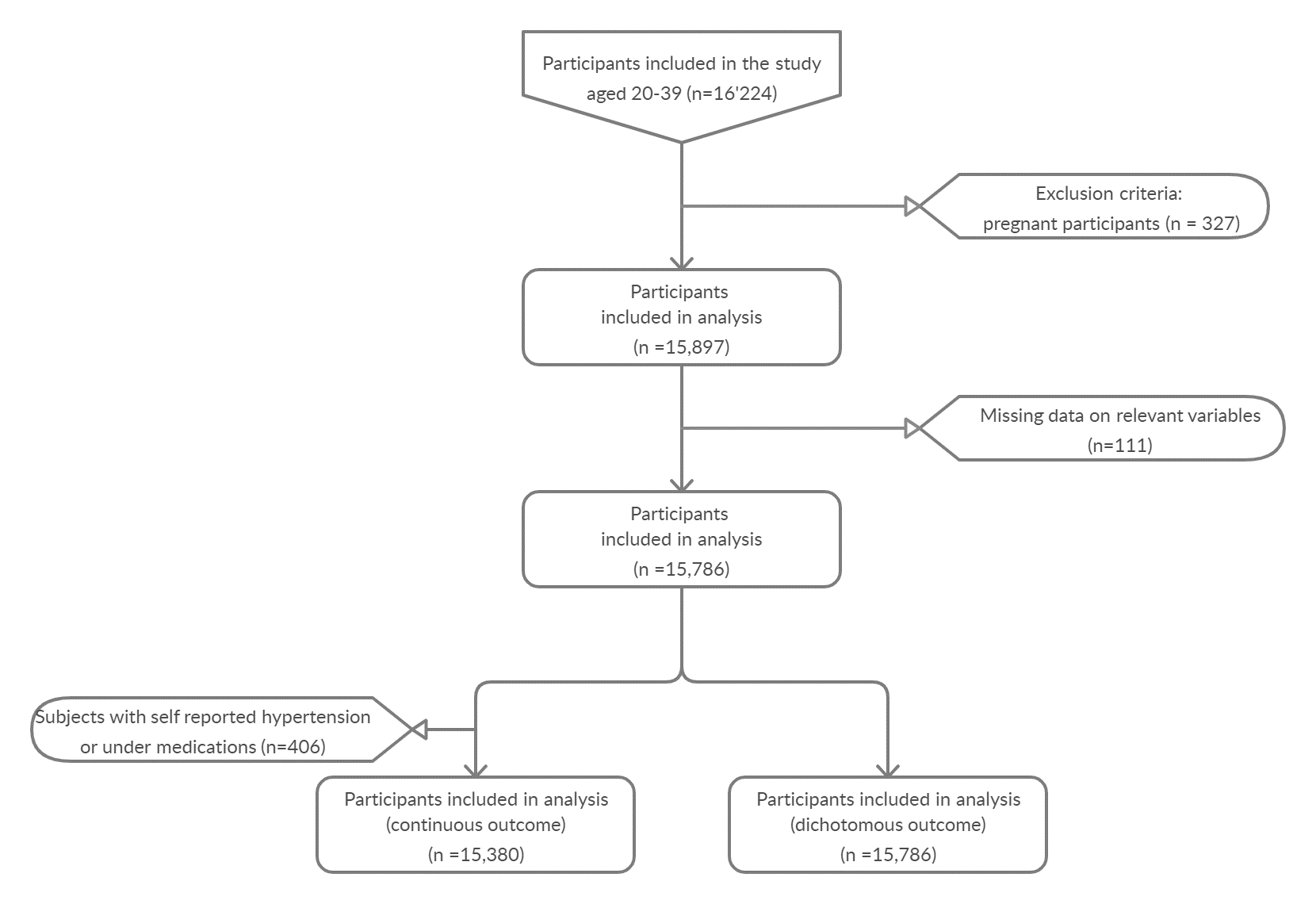

Supplement: Supplementary file 1 — Additional file 1: Figure 1. Flowchart of the study population. [file 12940_2020_656_MOESM1_ESM.docx]
