# Supplementary material for: Perfluoroalkyl substances are associated with elevated blood pressure and hypertension in highly exposed young adults
Source: Environ Health. 2020 Sep 21;19:102. doi: 10.1186/s12940-020-00656-0 (PMC7507812; doi:10.1186/s12940-020-00656-0)
Supplement: Supplementary file 2 — Additional file 2: Table 1. Descriptive statistics of diet and center where Blood Pressure has been measured. [file 12940_2020_656_MOESM2_ESM.docx]

**Additional File 2**

Table 1. Descriptive statistics of diet and center where Blood Pressure has been measured.

| **Covariates** | **Total - n (%)** | **M - n (%)** | **F - n (%)** |
| --- | --- | --- | --- |
| Fruit | | | |
| I Q | 4180 (26.48) | 2562 (33.42) | 1618 (19.93) |
| II Q | 5073 (32.14) | 2554 (33.31) | 2519 (31.03) |
| III Q | 3477 (22.03) | 1407 (18.35) | 2070 (25.5) |
| IV Q | 3056 (19.36) | 1144 (14.92) | 1912 (23.55) |
| Milk | | | |
| I Q | 4824 (30.56) | 2529 (32.99) | 2295 (28.27) |
| II Q | 3223 (20.42) | 1517 (19.79) | 1706 (21.01) |
| III Q | 6424 (40.69) | 3003 (39.17) | 3421 (42.14) |
| IV Q | 1315 (8.33) | 618 (8.06) | 697 (8.58) |
| Cheese | | | |
| I Q | 4699 (29.77) | 2041 (26.62) | 2658 (32.74) |
| II Q | 4042 (25.6) | 1819 (23.73) | 2223 (27.38) |
| III Q | 4193 (26.56) | 2213 (28.86) | 1980 (24.39) |
| IV Q | 2852 (18.07) | 1594 (20.79) | 1258 (15.49) |
| Meat | | | |
| I Q | 6561 (41.56) | 2438 (31.8) | 4123 (50.78) |
| II Q | 2717 (17.21) | 1307 (17.05) | 1410 (17.37) |
| III Q | 5658 (35.84) | 3301 (43.05) | 2357 (29.03) |
| IV Q | 850 (5.38) | 621 (8.1) | 229 (2.82) |
| Sweets/Snacks/Sweet beverages | | | |
| I Q | 5983 (37.9) | 3041 (39.66) | 2942 (36.24) |
| II Q | 4579 (29.01) | 2067 (26.96) | 2512 (30.94) |
| III Q | 3653 (23.14) | 1852 (24.16) | 1801 (22.18) |
| IV Q | 1571 (9.95) | 707 (9.22) | 864 (10.64) |
| Eggs | | | |
| I Q | 4532 (28.71) | 2077 (27.09) | 2455 (30.24) |
| II Q | 6531 (41.37) | 2969 (38.72) | 3562 (43.87) |
| III Q | 3157 (20) | 1635 (21.33) | 1522 (18.75) |
| IV Q | 1566 (9.92) | 986 (12.86) | 580 (7.14) |
| Fish | | | |
| I Q | 9395 (59.51) | 4591 (59.88) | 4804 (59.17) |
| II Q | 4184 (26.5) | 2027 (26.44) | 2157 (26.57) |
| III Q | 2207 (13.98) | 1049 (13.68) | 1158 (14.26) |
| Bread/Pasta/Cereals | | | |
| I Q | 7357 (46.6) | 3279 (42.77) | 4078 (50.23) |
| II Q | 6573 (41.64) | 3431 (44.75) | 3142 (38.7) |
| III Q | 1856 (11.76) | 957 (12.48) | 899 (11.07) |
| Salt | | | |
| Basso | 6650 (42.13) | 2941 (38.36) | 3709 (45.68) |
| Medio | 8243 (52.22) | 4256 (55.51) | 3987 (49.11) |
| Elevato | 893 (5.66) | 470 (6.13) | 423 (5.21) |
| Center | |  |  |
| Lonigo | 4067 (25.76) | 1972 (25.72) | 2095 (25.8) |
| Legnago | 3944 (24.98) | 1907 (24.87) | 2037 (25.09) |
| San Bonifacio | 4085 (25.88) | 1951 (25.45) | 2134 (26.28) |
| Noventa Vicentina | 3690 (23.38) | 1837 (23.96) | 1853 (22.82) |
