# Supplementary material for: Perfluoroalkyl substances are associated with elevated blood pressure and hypertension in highly exposed young adults
Source: Environ Health. 2020 Sep 21;19:102. doi: 10.1186/s12940-020-00656-0 (PMC7507812; doi:10.1186/s12940-020-00656-0)
Supplement: Supplementary file 3 — Additional file 3: Table 2. Descriptive statistics in subjects with or without hypertension, stratified by gender. [file 12940_2020_656_MOESM3_ESM.docx]

**Additional File 3**

Table 2. Descriptive statistics in subjects with or without hypertension, stratified by gender.

| *Continuous covariates* | | | | | | | | | | | | | | | |
| --- | --- | --- | --- | --- | --- | --- | --- | --- | --- | --- | --- | --- | --- | --- | --- |
|  | Total | | | | | M | | | | | F | | | | |
|  | Not Hypertensive | | Hypertensive | |  | Not Hypertensive | | Hypertensive | |  | Not Hypertensive | | Hypertensive | |  |
|  | mean | sd | mean | sd | p-value | mean | sd | mean | sd | p-value | mean | sd | mean | sd | p-value |
| Age | 29.93 | 5.86 | 30.86 | 5.83 | 0.000 | 29.73 | 5.88 | 30.49 | 5.81 | 0.000 | 30.08 | 5.83 | 31.88 | 5.74 | 0.000 |
| Time-lag | 14.72 | 5.48 | 15.10 | 5.23 | 0.004 | 14.65 | 5.57 | 14.88 | 5.26 | 0.150 | 14.78 | 5.41 | 15.71 | 5.12 | 0.000 |
| BMI | 23.49 | 3.96 | 26.87 | 5.29 | 0.000 | 24.28 | 3.40 | 26.81 | 4.73 | 0.000 | 22.84 | 4.25 | 27.02 | 6.62 | 0.000 |
| *Categorical covariates* | | | | | | | | | | | | | | | |
|  | NO | | YES | |  | NO | | YES | |  | NO | | YES | |  |
|  | Freq. | % | Freq. | % | p-value | Freq. | % | Freq. | % | p-value | Freq. | % | Freq. | % | p-value |
| **Gender** | | | | | | | | | | | | | | | |
| M | 6,218 | 45.01 | 1,449 | 73.52 | 0.000 |  | | | | | | | | | |
| F | 7,597 | 54.99 | 522 | 26.48 |  |  |  |  |  |  |  |  |  |  |  |
| **Smoke** | | | | | | | | | | | | | | | |
| NO | 8,174 | 59 | 1,056 | 53.58 | 0.000 | 3,182 | 51.17 | 713 | 49.21 | 0.370 | 4,992 | 65.71 | 343 | 65.71 | 0.774 |
| YES | 3,738 | 27.06 | 617 | 31.3 |  | 2,068 | 33.26 | 507 | 34.99 |  | 1,670 | 21.98 | 110 | 21.07 |  |
| Ex smoker | 1,903 | 13.77 | 298 | 15.12 |  | 968 | 15.57 | 229 | 15.8 |  | 935 | 12.31 | 69 | 13.22 |  |
| **Education** | | | | | | | | | | | | | | | |
| Elementary/Middle school | 2,038 | 14.75 | 371 | 18.82 | 0.000 | 1,063 | 17.1 | 283 | 19.53 | 0.000 | 975 | 12.83 | 88 | 16.86 | 0.000 |
| Highschool | 8,190 | 59.28 | 1,255 | 63.67 |  | 3,909 | 62.87 | 938 | 64.73 |  | 4,281 | 56.35 | 317 | 60.73 |  |
| University | 3,587 | 25.96 | 345 | 17.5 |  | 1,246 | 20.04 | 228 | 15.73 |  | 2,341 | 30.81 | 117 | 22.41 |  |
| **Physical activity** | | | | | | | | | | | | | | | |
| Light | 9,374 | 67.85 | 1,264 | 64.13 | 0.003 | 3,843 | 61.8 | 876 | 60.46 | 0.034 | 5,531 | 72.81 | 388 | 74.33 | 0.048 |
| Moderate | 2,056 | 14.88 | 338 | 17.15 |  | 903 | 14.52 | 249 | 17.18 |  | 1,153 | 15.18 | 89 | 17.05 |  |
| Heavy | 2,385 | 17.26 | 369 | 18.72 |  | 1,472 | 23.67 | 324 | 22.36 |  | 913 | 12.02 | 45 | 8.62 |  |
| **Country of birth** | | | | | | | | | | | | | | | |
| HDC | ##### | 90.38 | 1,811 | 91.88 | 0.033 | 5,773 | 92.84 | 1,363 | 94.06 | 0.099 | 6,713 | 88.36 | 448 | 85.82 | 0.082 |
| HMPC | 1,329 | 9.62 | 160 | 8.12 |  | 445 | 7.16 | 86 | 5.94 |  | 884 | 11.64 | 74 | 14.18 |  |
| **Fruit/Vegetables** | | | | | | | | | | | | | | | |
| 1 | 3,608 | 26.12 | 572 | 29.02 | 0.000 | 2,096 | 33.71 | 466 | 32.16 | 0.231 | 1,512 | 19.9 | 106 | 20.31 | 0.148 |
| 2 | 4,393 | 31.8 | 680 | 34.5 |  | 2,050 | 32.97 | 504 | 34.78 |  | 2,343 | 30.84 | 176 | 33.72 |  |
| 3 | 3,060 | 22.15 | 417 | 21.16 |  | 1,128 | 18.14 | 279 | 19.25 |  | 1,932 | 25.43 | 138 | 26.44 |  |
| 4 | 2,754 | 19.93 | 302 | 15.32 |  | 944 | 15.18 | 200 | 13.8 |  | 1,810 | 23.83 | 102 | 19.54 |  |
| **Milk/Yogurt** | | | | | | | | | | | | | | | |
| 1 | 4,180 | 30.26 | 644 | 32.67 | 0.057 | 2,036 | 32.74 | 493 | 34.02 | 0.580 | 2,144 | 28.22 | 151 | 28.93 | 0.525 |
| 2 | 2,808 | 20.33 | 415 | 21.06 |  | 1,222 | 19.65 | 295 | 20.36 |  | 1,586 | 20.88 | 120 | 22.99 |  |
| 3 | 5,661 | 40.98 | 763 | 38.71 |  | 2,452 | 39.43 | 551 | 38.03 |  | 3,209 | 42.24 | 212 | 40.61 |  |
| 4 | 1,166 | 8.44 | 149 | 7.56 |  | 508 | 8.17 | 110 | 7.59 |  | 658 | 8.66 | 39 | 7.47 |  |
| **Cheese** | | | | | | | | | | | | | | | |
| 1 | 4,163 | 30.13 | 536 | 27.19 | 0.049 | 1,672 | 26.89 | 369 | 25.47 | 0.729 | 2,491 | 32.79 | 167 | 31.99 | 0.305 |
| 2 | 3,528 | 25.54 | 514 | 26.08 |  | 1,466 | 23.58 | 353 | 24.36 |  | 2,062 | 27.14 | 161 | 30.84 |  |
| 3 | 3,654 | 26.45 | 539 | 27.35 |  | 1,791 | 28.8 | 422 | 29.12 |  | 1,863 | 24.52 | 117 | 22.41 |  |
| 4 | 2,470 | 17.88 | 382 | 19.38 |  | 1,289 | 20.73 | 305 | 21.05 |  | 1,181 | 15.55 | 77 | 14.75 |  |
| **Meat** | | | | | | | | | | | | | | | |
| 1 | 5,821 | 42.14 | 740 | 37.54 | 0.000 | 1,964 | 31.59 | 474 | 32.71 | 0.177 | 3,857 | 50.77 | 266 | 50.96 | 0.890 |
| 2 | 2,387 | 17.28 | 330 | 16.74 |  | 1,062 | 17.08 | 245 | 16.91 |  | 1,325 | 17.44 | 85 | 16.28 |  |
| 3 | 4,905 | 35.5 | 753 | 38.2 |  | 2,705 | 43.5 | 596 | 41.13 |  | 2,200 | 28.96 | 157 | 30.08 |  |
| 4 | 702 | 5.08 | 148 | 7.51 |  | 487 | 7.83 | 134 | 9.25 |  | 215 | 2.83 | 14 | 2.68 |  |
| **Sweets/Snacks/Sweet beverages** | | | | | | | | | | | | | | | |
| 1 | 5,186 | 37.54 | 797 | 40.44 | 0.103 | 2,439 | 39.22 | 602 | 41.55 | 0.302 | 2,747 | 36.16 | 195 | 37.36 | 0.843 |
| 2 | 4,032 | 29.19 | 547 | 27.75 |  | 1,685 | 27.1 | 382 | 26.36 |  | 2,347 | 30.89 | 165 | 31.61 |  |
| 3 | 3,216 | 23.28 | 437 | 22.17 |  | 1,524 | 24.51 | 328 | 22.64 |  | 1,692 | 22.27 | 109 | 20.88 |  |
| 4 | 1,381 | 10 | 190 | 9.64 |  | 570 | 9.17 | 137 | 9.45 |  | 811 | 10.68 | 53 | 10.15 |  |
| **Eggs** | | | | | | | | | | | | | | | |
| 1 | 3,918 | 28.36 | 614 | 31.15 | 0.034 | 1,655 | 26.62 | 422 | 29.12 | 0.049 | 2,263 | 29.79 | 192 | 36.78 | 0.003 |
| 2 | 5,734 | 41.51 | 797 | 40.44 |  | 2,394 | 38.5 | 575 | 39.68 |  | 3,340 | 43.96 | 222 | 42.53 |  |
| 3 | 2,797 | 20.25 | 360 | 18.26 |  | 1,351 | 21.73 | 284 | 19.6 |  | 1,446 | 19.03 | 76 | 14.56 |  |
| 4 | 1,366 | 9.89 | 200 | 10.15 |  | 818 | 13.16 | 168 | 11.59 |  | 548 | 7.21 | 32 | 6.13 |  |
| **Fish** | | | | | | | | | | | | | | | |
| 1 | 8,219 | 59.49 | 1,176 | 59.67 | 0.554 | 3,733 | 60.04 | 858 | 59.21 | 0.692 | 4,486 | 59.05 | 318 | 60.92 | 0.517 |
| 2 | 3,650 | 26.42 | 534 | 27.09 |  | 1,631 | 26.23 | 396 | 27.33 |  | 2,019 | 26.58 | 138 | 26.44 |  |
| 3 | 1,946 | 14.09 | 261 | 13.24 |  | 854 | 13.73 | 195 | 13.46 |  | 1,092 | 14.37 | 66 | 12.64 |  |
| **Bread/Pasta/Cereals** | | | | | | | | | | | | | | | |
| 1 | 6,437 | 46.59 | 920 | 46.68 | 0.753 | 2,634 | 42.36 | 645 | 44.51 | 0.225 | 3,803 | 50.06 | 275 | 52.68 | 0.504 |
| 2 | 5,744 | 41.58 | 829 | 42.06 |  | 2,793 | 44.92 | 638 | 44.03 |  | 2,951 | 38.84 | 191 | 36.59 |  |
| 3 | 1,634 | 11.83 | 222 | 11.26 |  | 791 | 12.72 | 166 | 11.46 |  | 843 | 11.1 | 56 | 10.73 |  |
| **Alcohol consumption** | | | | | | | | | | | | | | | |
| NO | 3,364 | 24.35 | 380 | 19.28 | 0.000 | 831 | 13.36 | 169 | 11.66 | 0.063 | 2,533 | 33.34 | 211 | 40.42 | 0.004 |
| less than e per week | 5,684 | 41.14 | 731 | 37.09 |  | 2,218 | 35.67 | 509 | 35.13 |  | 3,466 | 45.62 | 222 | 42.53 |  |
| 3-6 per week | 2,991 | 21.65 | 472 | 23.95 |  | 1,809 | 29.09 | 412 | 28.43 |  | 1,182 | 15.56 | 60 | 11.49 |  |
| more than 7 per week | 1,776 | 12.86 | 388 | 19.69 |  | 1,360 | 21.87 | 359 | 24.78 |  | 416 | 5.48 | 29 | 5.56 |  |
| **Salt consumption** | | | | | | | | | | | | | | | |
| Basso | 5,812 | 42.07 | 838 | 42.52 | 0.893 | 2,363 | 38 | 578 | 39.89 | 0.373 | 3,449 | 45.4 | 260 | 49.81 | 0.148 |
| Medio | 7,218 | 52.25 | 1,025 | 52 |  | 3,468 | 55.77 | 788 | 54.38 |  | 3,750 | 49.36 | 237 | 45.4 |  |
| Elevato | 785 | 5.68 | 108 | 5.48 |  | 387 | 6.22 | 83 | 5.73 |  | 398 | 5.24 | 25 | 4.79 |  |
| **Center where Blood Pressure have been measured** | | | | | | | | | | | | | | | |
| Lonigo | 3,539 | 25.62 | 528 | 26.79 | 0.000 | 1,575 | 25.33 | 397 | 27.4 | 0.000 | 1,964 | 25.85 | 131 | 25.1 | 0.000 |
| Legnago | 3,368 | 24.38 | 576 | 29.22 |  | 1,494 | 24.03 | 413 | 28.5 |  | 1,874 | 24.67 | 163 | 31.23 |  |
| San Bonifacio | 3,544 | 25.65 | 541 | 27.45 |  | 1,554 | 24.99 | 397 | 27.4 |  | 1,990 | 26.19 | 144 | 27.59 |  |
| Noventa Vicentina | 3,364 | 24.35 | 326 | 16.54 |  | 1,595 | 25.65 | 242 | 16.7 |  | 1,769 | 23.29 | 84 | 16.09 |  |
