# Supplementary material for: Perfluoroalkyl substances are associated with elevated blood pressure and hypertension in highly exposed young adults
Source: Environ Health. 2020 Sep 21;19:102. doi: 10.1186/s12940-020-00656-0 (PMC7507812; doi:10.1186/s12940-020-00656-0)
Supplement: Supplementary file 5 — Additional file 5: Figure 3. Predicted values of Systolic and Diastolic Blood Pressure by deciles of PFAS distribution. [file 12940_2020_656_MOESM5_ESM.docx]

**Additional File 5**

Figure 3. Exposure–response curves for PFAS exposure and Systolic and Diastolic Blood pressure from GAM models using thin plane splines, with 95% confidence intervals, stratified by gender. The predicted levels are based on average characteristics used as covariates in the models.

| \| SYSTOLIC BLOOD PRESSURE \| DIASTOLIC BLOOD PRESSURE \| \| --- \| --- \| \| 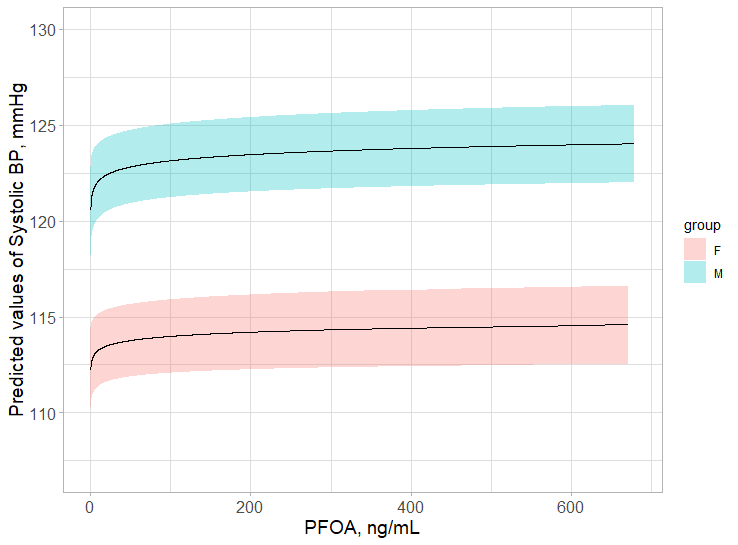 \| 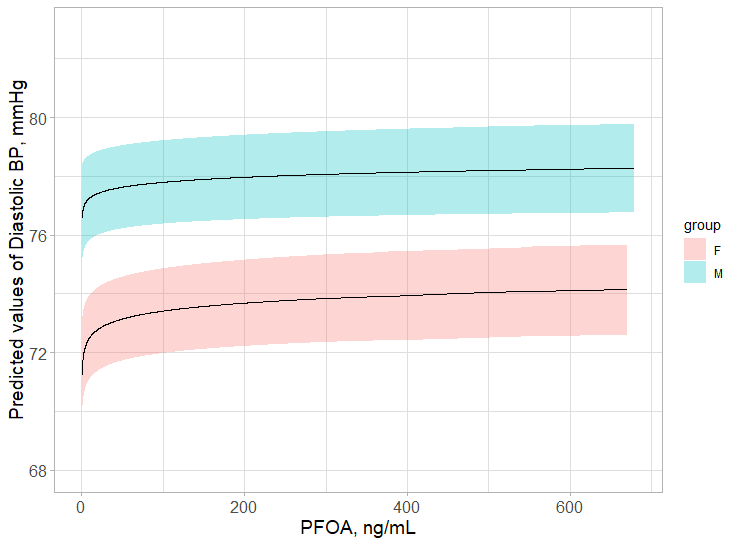 \| \| 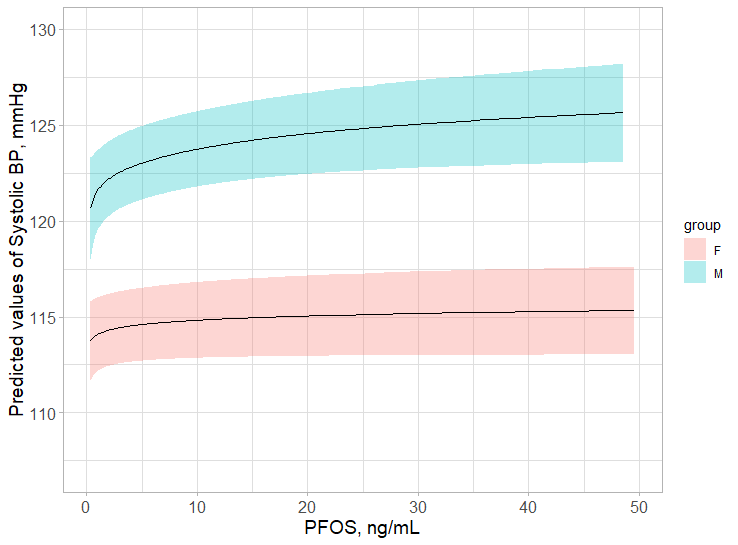 \| 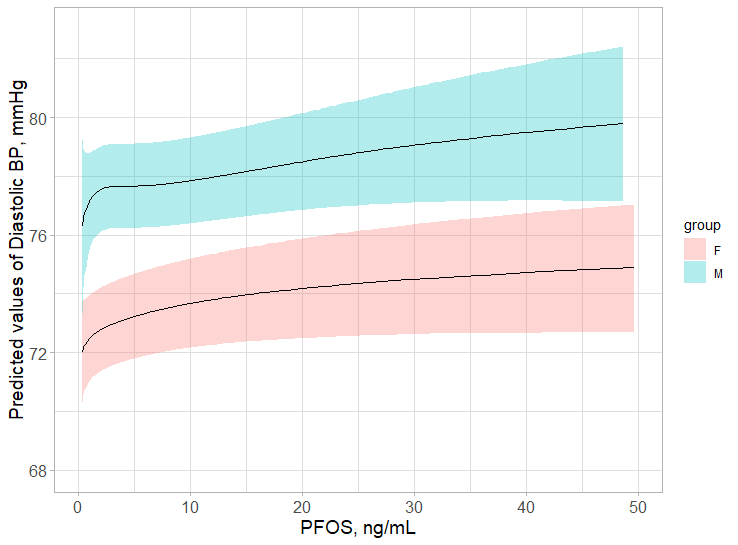 \| \| 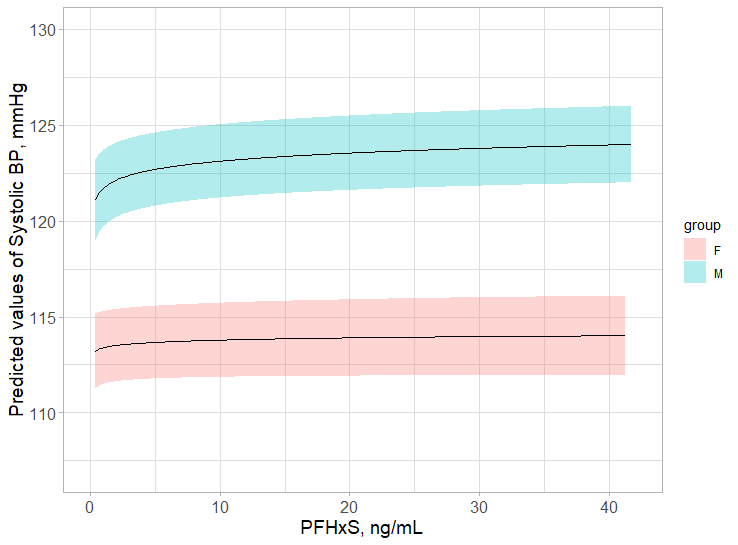 \| 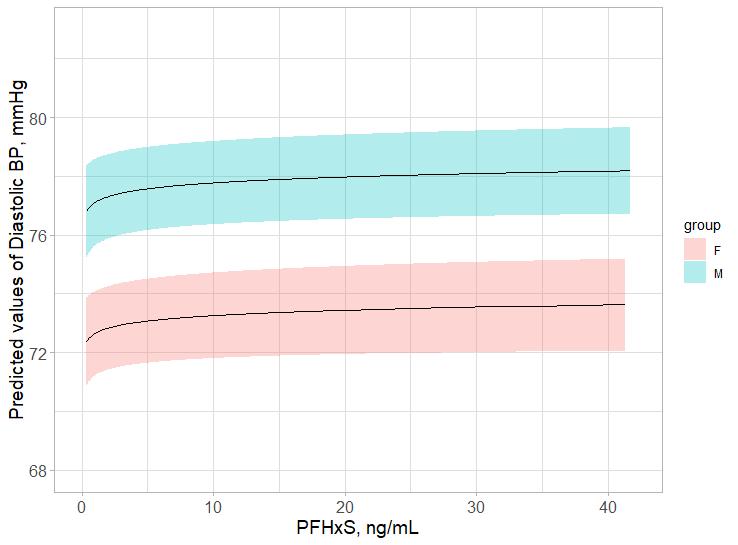 \| \| 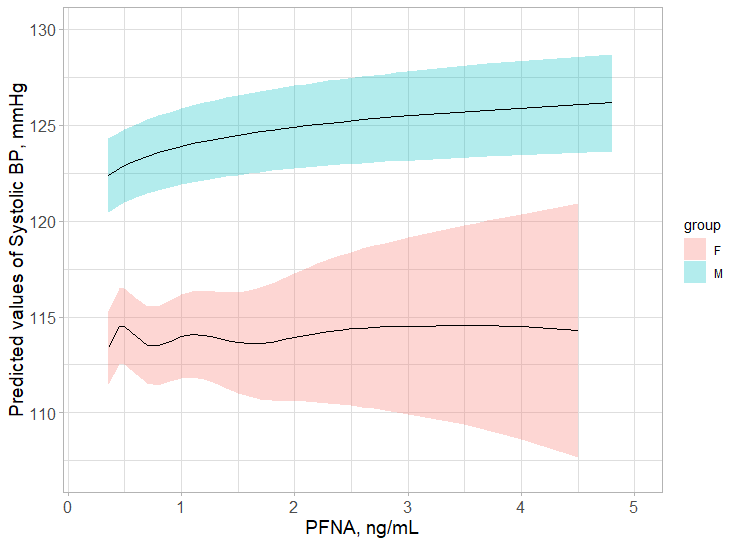 \| 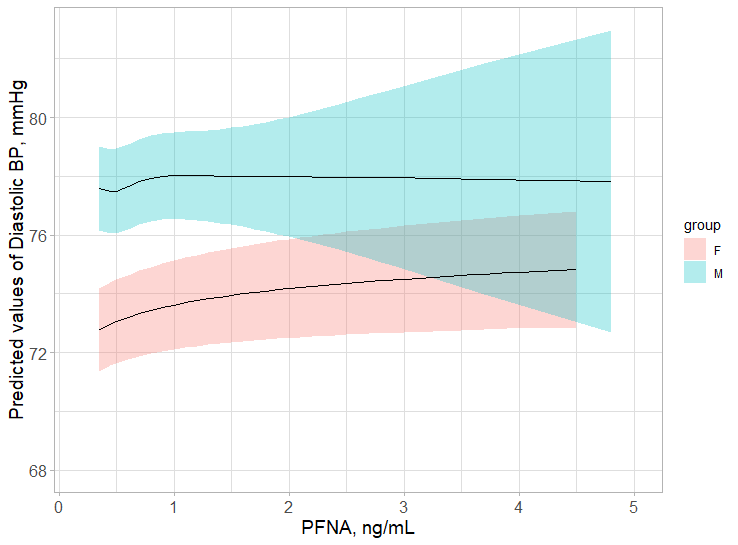 \| |  |
| --- | --- | --- | --- | --- | --- | --- | --- | --- | --- | --- | --- |
