# Supplementary material for: Perfluoroalkyl substances are associated with elevated blood pressure and hypertension in highly exposed young adults
Source: Environ Health. 2020 Sep 21;19:102. doi: 10.1186/s12940-020-00656-0 (PMC7507812; doi:10.1186/s12940-020-00656-0)
Supplement: Supplementary file 6 — Additional file 6: Table 3. GAM model with using PFAS thin plate spline smooth terms: EDF and p-values. [file 12940_2020_656_MOESM6_ESM.docx]

**Additional File 6**

Table 3. GAM model with PFAS thin plate spline smooth terms: EDF and p-values.

|  | **Systolic Blood Pressure** | | | | | | **Diastolic Blood Pressure** | | | | | | **Hypertension** | | | | | |
| --- | --- | --- | --- | --- | --- | --- | --- | --- | --- | --- | --- | --- | --- | --- | --- | --- | --- | --- |
| **PFAS** | **Total** | | **Males** | | **Females** | | **Total** | | **Males** | | **Females** | | **Total** | | **Males** | | **Females** | |
|  | **EDF** | **P-values** | **EDF** | **P-values** | **EDF** | **P-values** | **EDF** | **P-values** | **EDF** | **P-values** | **EDF** | **P-values** | **EDF** | **P-values** | **EDF** | **P-values** | **EDF** | **P-values** |
| log_PFOA | **1.00** | 0.000 | **1.00** | 0.001 | **1.00** | 0.010 | **1.00** | 0.000 | **1.10** | 0.023 | **1.00** | 0.000 | **1.99** | 0.020 | **2.41** | 0.018 | 5.02 | 0.247 |
| log_PFOS | **3.06** | 0.003 | **1.34** | 0.001 | 1.00 | 0.159 | **3.12** | 0.004 | 2.76 | 0.228 | **1.47** | 0.011 | **1.00** | 0.016 | **1.00** | 0.005 | 2.82 | 0.424 |
| log_PFHxS | **1.46** | 0.003 | **1.00** | 0.000 | 1.00 | 0.263 | **1.00** | 0.000 | **1.00** | 0.011 | **1.00** | 0.023 | **1.00** | 0.005 | **1.00** | 0.003 | 2.61 | 0.308 |
| log_PFNA | **1.00** | 0.000 | **1.00** | 0.000 | 4.87 | 0.084 | **1.00** | 0.001 | 2.70 | 0.366 | **1.00** | 0.005 | 1.00 | 0.164 | **1.00** | 0.029 | 1.13 | 0.759 |
