# Supplementary material for: Perfluoroalkyl substances are associated with elevated blood pressure and hypertension in highly exposed young adults
Source: Environ Health. 2020 Sep 21;19:102. doi: 10.1186/s12940-020-00656-0 (PMC7507812; doi:10.1186/s12940-020-00656-0)
Supplement: Supplementary file 7 — Additional file 7: Table 4. GAM models adjusted by eGFR (cut-off 90 ml/min). [file 12940_2020_656_MOESM7_ESM.docx]

**Additional File 7**

Table 4. GAM models adjusted by eGFR (cut-off 90 ml/min).

| **PFAS** | **Systolic Blood Pressure** | | | | | | **Diastolic Blood Pressure** | | | | | |
| --- | --- | --- | --- | --- | --- | --- | --- | --- | --- | --- | --- | --- |
|  | **Total** | | **Males** | | **Females** | | **Total** | | **Males** | | **Females** | |
|  | **β (CI 95%)** | **p-value** | **β (CI 95%)** | **p-value** | **β (CI 95%)** | **p-value** | **β (CI 95%)** | **p-value** | **β (CI 95%)** | **p-value** | **β (CI 95%)** | **p-value** |
| ln_PFOA | 0.37 (0.19-0.55) | 0.000 | 0.46 (0.19-0.73) | 0.001 | 0.32 (0.08-0.56) | 0.008 | 0.35 (0.22-0.48) | 0.000 | 0.23 (0.04-0.42) | 0.016 | 0.39 (0.21-0.57) | 0.000 |
| IQ | 113.8 (0-0) |  | 122.11 (0-0) |  | 113.02 (0-0) |  | 73.25 (0-0) |  | 77.39 (0-0) |  | 72.33 (0-0) |  |
| II Q | 0.26 (-0.29-0.81) | 0.359 | 0.12 (-0.87-1.1) | 0.818 | 0.47 (-0.19-1.13) | 0.164 | 0.24 (-0.16-0.64) | 0.240 | -0.31 (-1-0.39) | 0.385 | 0.45 (-0.05-0.95) | 0.080 |
| III Q | 0.74 (0.16-1.31) | 0.012 | 0.8 (-0.16-1.75) | 0.101 | 0.81 (0.07-1.54) | 0.031 | 0.78 (0.36-1.2) | 0.000 | 0.4 (-0.27-1.07) | 0.244 | 0.75 (0.19-1.3) | 0.008 |
| IV Q | 1.07 (0.46-1.69) | 0.001 | 1.25 (0.31-2.18) | 0.009 | 0.73 (-0.18-1.64) | 0.115 | 0.98 (0.53-1.43) | 0.000 | 0.49 (-0.17-1.14) | 0.145 | 1.16 (0.47-1.84) | 0.001 |
| ln_PFOS | 0.57 (0.24-0.91) | 0.001 | 0.97 (0.47-1.48) | 0.000 | 0.33 (-0.11-0.78) | 0.145 | 0.44 (0.2-0.69) | 0.000 | 0.29 (-0.07-0.65) | 0.110 | 0.51 (0.18-0.85) | 0.003 |
| IQ | 114.14 (0-0) |  | 122.24 (0-0) |  | 114.26 (0-0) |  | 73.48 (0-0) |  | 77.5 (0-0) |  | 72.63 (0-0) |  |
| II Q | -0.01 (-0.56-0.54) | 0.970 | 0.46 (-0.55-1.47) | 0.368 | -0.06 (-0.71-0.59) | 0.851 | 0.32 (-0.08-0.72) | 0.114 | 0.3 (-0.41-1.01) | 0.408 | 0.25 (-0.24-0.74) | 0.317 |
| III Q | 0.28 (-0.29-0.84) | 0.341 | 0.79 (-0.18-1.76) | 0.110 | 0.27 (-0.46-0.99) | 0.474 | 0.3 (-0.11-0.71) | 0.156 | 0.12 (-0.57-0.8) | 0.736 | 0.26 (-0.29-0.81) | 0.348 |
| IV Q | 0.61 (0-1.21) | 0.050 | 1.12 (0.15-2.08) | 0.023 | 0.44 (-0.44-1.31) | 0.327 | 0.58 (0.14-1.02) | 0.010 | 0.19 (-0.49-0.87) | 0.577 | 1.01 (0.35-1.67) | 0.003 |
| ln_PFHxS | 0.37 (0.15-0.58) | 0.001 | 0.61 (0.29-0.92) | 0.000 | 0.18 (-0.12-0.48) | 0.239 | 0.33 (0.17-0.49) | 0.000 | 0.29 (0.07-0.51) | 0.011 | 0.27 (0.04-0.49) | 0.021 |
| IQ | 113.97 (0-0) |  | 121.73 (0-0) |  | 113.33 (0-0) |  | 73.34 (0-0) |  | 77.02 (0-0) |  | 72.61 (0-0) |  |
| II Q | -0.01 (-0.55-0.54) | 0.982 | 0.63 (-0.43-1.69) | 0.247 | -0.13 (-0.77-0.51) | 0.687 | 0.21 (-0.19-0.61) | 0.312 | 0.5 (-0.25-1.24) | 0.193 | 0.04 (-0.44-0.53) | 0.863 |
| III Q | 0.61 (0.04-1.18) | 0.036 | 1.16 (0.14-2.17) | 0.025 | 0.58 (-0.13-1.29) | 0.111 | 0.7 (0.29-1.12) | 0.001 | 0.74 (0.03-1.46) | 0.041 | 0.58 (0.04-1.11) | 0.035 |
| IV Q | 0.94 (0.3-1.57) | 0.004 | 1.54 (0.54-2.53) | 0.002 | 0.46 (-0.58-1.49) | 0.387 | 0.79 (0.33-1.25) | 0.001 | 0.75 (0.05-1.45) | 0.036 | 0.76 (-0.02-1.55) | 0.055 |
| ln_PFNA | 0.99 (0.47-1.51) | 0.000 | 1.45 (0.71-2.18) | 0.000 | 0.55 (-0.19-1.29) | 0.147 | 0.63 (0.25-1.01) | 0.001 | 0.36 (-0.15-0.88) | 0.169 | 0.81 (0.25-1.38) | 0.004 |
