# Supplementary material for: Perfluoroalkyl substances are associated with elevated blood pressure and hypertension in highly exposed young adults
Source: Environ Health. 2020 Sep 21;19:102. doi: 10.1186/s12940-020-00656-0 (PMC7507812; doi:10.1186/s12940-020-00656-0)
Supplement: Supplementary file 8 — Additional file 8: Table 5. GAM models on a restricted population recruited after December 2017 (n = 10,656). [file 12940_2020_656_MOESM8_ESM.docx]

**Additional File 8**

Table 5. GAM models on a restricted population recruited after December 2017 (n=10,656).

| **PFAS** | **Systolic Blood Pressure** | | | | | | **Diastolic Blood Pressure** | | | | | |
| --- | --- | --- | --- | --- | --- | --- | --- | --- | --- | --- | --- | --- |
|  | **Total** | | **Males** | | **Females** | | **Total** | | **Males** | | **Females** | |
|  | **β (CI 95%)** | **p-value** | **β (CI 95%)** | **p-value** | **β (CI 95%)** | **p-value** | **β (CI 95%)** | **p-value** | **β (CI 95%)** | **p-value** | **β (CI 95%)** | **p-value** |
| ln_PFOA | 0.32 (0.12-0.52) | 0.002 | 0.5 (0.21-0.8) | 0.001 | 0.15 (-0.12-0.42) | 0.271 | 0.3 (0.16-0.45) | 0.000 | 0.21 (0-0.42) | 0.051 | 0.35 (0.14-0.56) | 0.001 |
| ln_PFOS | 0.56 (0.16-0.95) | 0.005 | 0.89 (0.3-1.48) | 0.003 | 0.28 (-0.24-0.81) | 0.292 | 0.39 (0.1-0.68) | 0.008 | 0.27 (-0.15-0.69) | 0.207 | 0.46 (0.05-0.86) | 0.027 |
| ln_PFHxS | 0.36 (0.11-0.6) | 0.004 | 0.67 (0.31-1.02) | 0.000 | 0.01 (-0.34-0.35) | 0.959 | 0.29 (0.11-0.47) | 0.002 | 0.3 (0.05-0.56) | 0.018 | 0.21 (-0.06-0.47) | 0.124 |
| ln_PFNA | 0.79 (0.17-1.4) | 0.013 | 1.28 (0.42-2.14) | 0.004 | 0.17 (-0.74-1.07) | 0.720 | 0.6 (0.14-1.06) | 0.010 | 0.28 (-0.33-0.9) | 0.368 | 0.84 (0.14-1.54) | 0.018 |
