# Supplementary material for: Perfluoroalkyl substances are associated with elevated blood pressure and hypertension in highly exposed young adults
Source: Environ Health. 2020 Sep 21;19:102. doi: 10.1186/s12940-020-00656-0 (PMC7507812; doi:10.1186/s12940-020-00656-0)
Supplement: Supplementary file 9 — Additional file 9: Table 6. GAM models on a restricted population, excluding subclinical hypertensive (n = 13,815). [file 12940_2020_656_MOESM9_ESM.docx]

**Additional File 9**

Table 6. GAM models on a restricted population, excluding subclinical hypertensive (n=13,815).

| **PFAS** | **Systolic Blood Pressure** | | | | | | **Diastolic Blood Pressure** | | | | | |
| --- | --- | --- | --- | --- | --- | --- | --- | --- | --- | --- | --- | --- |
|  | **Total** | | **Males** | | **Females** | | **Total** | | **Males** | | **Females** | |
|  | **β (CI 95%)** | **p-value** | **β (CI 95%)** | **p-value** | **β (CI 95%)** | **p-value** | **β (CI 95%)** | **p-value** | **β (CI 95%)** | **p-value** | **β (CI 95%)** | **p-value** |
| ln_PFOA | 0.3 (0.14-0.46) | 0.000 | 0.3 (0.06-0.53) | 0.013 | 0.28 (0.05-0.5) | 0.015 | 0.3 (0.18-0.43) | 0.000 | 0.17 (-0.01-0.36) | 0.060 | 0.34 (0.17-0.51) | 0.000 |
| ln_PFOS | 0.42 (0.12-0.72) | 0.006 | 0.52 (0.08-0.96) | 0.022 | 0.33 (-0.08-0.75) | 0.116 | 0.45 (0.22-0.68) | 0.000 | 0.28 (-0.06-0.62) | 0.105 | 0.5 (0.18-0.82) | 0.002 |
| ln_PFHxS | 0.3 (0.1-0.49) | 0.003 | 0.42 (0.14-0.69) | 0.003 | 0.15 (-0.13-0.43) | 0.294 | 0.28 (0.13-0.44) | 0.000 | 0.22 (0.01-0.43) | 0.044 | 0.24 (0.02-0.46) | 0.030 |
| ln_PFNA | 0.77 (0.3-1.24) | 0.001 | 0.79 (0.15-1.43) | 0.016 | 0.7 (0.01-1.39) | 0.046 | 0.66 (0.3-1.03) | 0.000 | 0.3 (-0.19-0.8) | 0.233 | 0.89 (0.36-1.42) | 0.001 |
